# Protective effects of total flavonoids from *Alpinia officinarum* rhizoma against ethanol-induced gastric ulcer *in vivo* and *in vitro*

**DOI:** 10.1080/13880209.2020.1803370

**Published:** 2020-09-01

**Authors:** Kaiwen Lin, Yong Wang, Jingwen Gong, Yinfeng Tan, Tang Deng, Na Wei

**Affiliations:** a Key Laboratory of Tropical Translational Medicine of Ministry of Education, Haikou, China; b Hainan Provincial Key Laboratory for Research and Development of Tropical Herbs, Haikou, China; c School of Pharmacy, Hainan Medical University, Haikou, China; d Intervention Vascular Surgery of First Affiliated Hospital of Hainan Medical University, Haikou, China; e Ministry of Education, Key Laboratory of Emergency and Trauma of Hainan Medical University, Haikou, China

**Keywords:** Gastroprotection, inflammatory cytokines, gastric cytoprotection, nitric oxide, NF-κB, COX-2, flavonoids

## Abstract

**Context:**

*Alpinia officinarum* Hance (Zingiberaceae) is traditionally used to treat inflammation, pain, colds and digestive diseases.

**Objective:**

To investigate the potential protective mechanism of total flavonoids from the rhizomes of *A. officinarum* (F-AOH) in ethanol-induced acute gastric *in vivo* and *in vitro*.

**Materials and methods:**

*In vivo*: Gastric damage was induced in BALB/c mice by administering ethanol (10 mL/kg) after oral treatment with F-AOH at 126.8, 63.4 and 31.7 mg/kg or ranitidine (Ran) at 100 mg/kg (1 week of continuous gavage). *In vitro*: Gastric mucosal epithelial cells (GES-1) were incubated with F-AOH (8, 4 and 2 μg/mL) for 16 h and treated with 7% ethanol for 4 h. The extent of gastric damage was assessed histopathologically, and the expression of NF-κB, COX-2, TNF-α, iNOS and IL-1β was quantified by Western blot analysis. In addition, proinflammatory mediators and concentrations of motilin (MTL) and gastrin (GAS) were measured by ELISA test.

**Results:**

F-AOH effectively reduced the ulcer index (from 23.4 ± 4.28 to 8.32 ± 1.5) and reduced release of inflammatory mediators (IL-1β, IL-6, TNF-α and PGE2), increased the content of nitric oxide and improved GAS and MTL secretion. The 50% inhibitory concentration (IC_50_) of F-AOH on cell damage was 17 μg/mL. F-AOH increased ethanol-induced cell survival (from 47 to 85%) and inhibited the expression of NF-κB, COX-2, TNF-α, IL-1β and iNOS proteins.

**Conclusions:**

F-AOH inhibits ethanol-induced gastric mucosal damage, provides a theoretical basis for galangal in the treatment of other causes of GU, and promotes the application of galanga in the treatment of GU.

## Introduction

At present, gastric ulcer has become a common digestive disease encountered clinically. There are several pathogenic and aggravating factors contributing to gastric ulcers, such as *Helicobacter pylori* infection (Peterson 1991), long-term use of non-steroidal anti-inflammatory drugs (Matsui et al. 2011), stressful mental states (Zhao et al. 2018), excessive gastric acid secretion (Wijeratne et al. 2015) and genetic predisposition (Tahara et al. 2007). The type of gastric ulcer induced by ethanol tends to erode the gastric tissue, causing extreme damage to the gastric mucosa, including haemorrhagic damage to the gastric mucosal lesions and mucosal oedema (Park et al. 2008), inflammatory cell infiltration (Xie et al. 2017) and diffuse ulcers (Al-Sayed and El-Naga 2015). NF-κB is one of the important transcription factors regulating the development of gastric ulcer. Its activation can regulate the immune response and the release and expression of proinflammatory mediators such as TNF-α, IL-1β, IL-6 and COX-2, thus aggravating ulcer injury (Chang et al. 2015; Chen et al. 2019). In ethanol-induced gastric ulcer, PGE2 was found to be associated with promoting the regeneration of gastric mucosal epithelial cells, repairing the ulcerous area and inhibiting gastric acid secretion. NO promotes the healing of gastric ulcer by improving mucosal microcirculation and scavenging oxygen free radicals (Elliott and Wallace 1998; Takeuchi et al. 2001; Yu et al. 2015). Moreover, ethanol exacerbates the extent of gastric ulcer by enhancing gastric contraction and small bowel movements, promoting gastric emptying and the pathogenesis of gastric acid secretion (Konturek et al. 1994).

*Alpinia officinarum* Hance (Zingiberaceae) is widely distributed in many tropical regions of Asia. In China, it is mainly distributed in Guangdong, Hainan and Yunnan. Its main chemical components are volatile oil, flavonoids and diarylheptanoids, which were found to treat digestive diseases such as indigestion, acid reflux and gastric ulcer. Many studies demonstrated its pharmacological activities, such as antibacterial (Zhang et al. 2010), antioxidant, (Ly et al. 2003), antitumor (Tabata et al. 2009) and anti-inflammatory effects (Lee et al. 2009). It has been proven that the extract of *A. officinarum* could be used as a beneficial medicine for ethanol-induced acute gastric injury (Wei et al. 2015) and indomethacin-induced gastric injury (Gong et al. 2018). In addition, it has been confirmed that the extract mainly contains galangin, kaempferol, 5-hydroxy-7-(4-hydroxy-3-methoxyphenyl)-1-phenyl-3-heptanone (DPHA), 7-(4-hydroxy-3-methoxyphenyl)-1-phenyl-4-ene-3-heptanone (DPHB) and 1,7-diphenyl-5-hydroxy-3-heptanone (DPHC). Among them, galangin and kaempferol are flavonoids. Therefore, we collected F-AOH by chemical separation and investigated its protective effect on ethanol-induced acute gastric injury, which provided a certain research basis on the effective components of F-AOH against gastric ulcer. Consequently, we evaluated the protective, healing and anti-inflammatory mechanisms of F-AOH in ethanol-induced acute gastric ulcer by conducting *in vivo/in vitro* experiments with histological and pathological examination and by using inflammatory factors as markers.

## Materials and methods

### Plant material

Rhizomes of *A. officinarum* were collected from Haikou County, Hainan Province, China, in October 2017. The plant was identified by Professor Niankai Zeng of Hainan Medical University. A voucher specimen of this collection (no. 20171024) has been deposited in the Laboratory of Natural Pharmaceutical Chemistry of Hainan Medical University. The plant materials were air-dried and weighed, which were then grounded into coarse powder by a grinder (XL-06A, Xu Machinery, Guangzhou, China).

### Preparation of plant extract

An aliquot (1 kg) of fresh *Alpinia officinarum* rhizomes was weighed precisely and refluxed with eightfold 80% ethanol for 1 h. The residue was extracted twice under the same conditions. The sampled ethanol extracts were combined and concentrated to 40% under reduced pressure. Then, the extract was purified with AB-8 macroporous resin by 80% ethanol. The ethanol elution fraction was subjected to silica gel column chromatography and eluted with a petroleum ether-ethyl acetate gradient to obtain six crude components (1–6), with components 4 and 5 being subjected to gel column chromatography and eluted with methanol.

### Phytochemical analysis

The ultra-high performance liquid chromatography-diode array detector-mass spectrometry (UHPLC-DAD-MS) data were obtained from an Agilent 1290 Infinity series UHPLC system with a diode array detector and an Agilent 6120 quadrupole mass spectrometer (Agilent Technologies, Santa Clara, CA). The mass spectrometer contained a dual atmospheric pressure chemical ionization (APCI) and electrospray ionization (ESI) interface. The liquid chromatography (LC) column was an Agilent ZORBAX Eclipse Plus C18 (2.1 × 100 mm, 1.8 μm). The column temperature was 30 °C. The flow rate was 0.25 mL/min. The mobile phase eluent consisted of isopropanol with 5 mM ammonium formate (B) and water (A), both containing 0.05% formic acid. The gradient was 10% B programmed to 80% in 20 min, and then programmed to 100% in 22 min. Ionization and detection of compounds were carried out on the mass spectrometer using the ESI positive mode over the mass range of *m/z* 100–800. The fragmentor and capillary voltages were 100 V and 4000 V, respectively. The drying gas flow rate was 12.0 L/min, the nebulizer pressure was 30 psi and the drying gas temperature was 300 °C. The DAD was employed to monitor at 254, 280 and 325 nm.

### Animals

A total of 60 BALB/c female mice (18–22 g) from Tianqin Biotechnology (Changsha, China) were housed under controlled conditions (12 h light/dark cycle and room temperature of 24 ± 1 °C and 40–60% relative humidity). They had free access to food and water. All animal experiments were carried out in accordance with the recommendations of the International Guidelines for the Use and Care of Experimental Animals and conducted with the permission from the Animal Experimental Ethics Committee of Hainan Medical University (no. hy2018031601, Haikou, China).

### Cell culture

Human gastric mucosal epithelial cells (GES-1) (Procell Life Science & Technology, Wuhan, China) were cultured at 37 °C under 5% CO_2_ in RPMI 1640 culture medium (Gibco, Brooklyn, NY) supplied with 10% heat-inactivated foetal bovine serum (FBS) (Biological Industries, Beit Haemek, Israel), 100 U/mL of penicillin and 100 μ/mL of streptomycin (Beyotime, Nanjing, China).

### Animal experiment design

After three days of adaptive feeding, the mice were randomly divided into six groups: normal group, ranitidine control group (100 mg/kg), ethanol group and F-AOH high- (126.8 mg/kg), medium- (63.4 mg/kg) and low-dose (31.7 mg/kg) groups. The F-AOH groups were all formulated with 0.5% sodium carboxymethylcellulose (CMC-Na). The normal group and the ethanol group were given the same volume of 0.5% CMC-Na, once a day for seven days. The mice were fasted for 24 h prior to the experiment, but allowed free access to water. Ulcer was induced with 100% ethanol (10 mL/kg). The normal control group received only 0.5% CMC-Na. After 1 h from induction, the mice were euthanized under deep anaesthesia with isoflurane. The stomach was removed and cut along the greater curvature, washed with ice-cold saline, placed on a white wax plate; the blood spots were photographed with a camera; and the ulcer injury index was assessed based on the area of haemorrhagic mucosal lesions. The gastric tissue was then divided into two parts: one part was placed in 4% paraformaldehyde solution for histological analysis, and the other part was stored at − 80 °C for biochemical analysis.

### Detection of cell vitality

GES-1 cells were seeded on a 96-well plate at a density of 1 × 10^4^ cells per well for 4 h and then treated with F-AOH (16, 8, 4, 2 and 1 µg/mL), while the normal group and the ethanol group were cultured in a serum-free medium, followed by incubation at 37 °C for 16 h. The normal group was stimulated with an equal volume of serum-free medium, while 7% alcohol was added and co-incubated for 4 h; 10 µL cell counting kit-8 (CCK-8) and 90 µL serum-free medium were added into each well. After incubation for 2 h in a cell culture incubator, the optical density (OD) value was then measured on a microplate reader at 450 nm. The cell viability was calculated according to the equation:

(1) Cell viability = [(As−Ab)/(Ac−Ab)]×100%

where As is the OD of the F-AOH group or OD of the ethanol group, Ab is the OD of the blank hole and Ac is the OD of the normal group.

### Determination of the injury index of gastric ulcer

The ulcer index (UI) was calculated according to the following equation:

(2) Ulcer index (UI)=(1A)+(2B)+(3C)

where *A* is the number of small ulcers ≤1 mm, *B*: 3 mm ≥ the number of ulcer >1 mm and *C* is the number of linear ulcers >3 mm.

The percentage of drug group inhibition was calculated according to equation:

(3) $$\text{Inhibition}=\left[\left({\text{UI}}^{1}-{\;\text{UI}}^{2}\right)\;\times \;100\%\right]/{\text{UI}}^{1}$$

where UI^1^ is the ulcer index of the ethanol group and UI^2^ is the ulcer index of the F-AOH group.

### Histopathological analysis

Gastric tissues removed from mice were fixed in 4% paraformaldehyde solution, dehydrated with ethanol and xylene, and embedded in paraffin. Paraffin sections were then cut to a thickness of 3 μm. These sections were stained with haematoxylin and eosin for histological evaluation.

### Enzyme-linked immunosorbent assay

The levels of proinflammatory cytokines TNF-α, IL-1β and IL-6 in gastric tissue and GES-1 supernatant were determined using an ELISA kit (Elisa Biotech, Shanghai, China). Simultaneously, the levels of PGE2, NO, motilin (MTL) and gastrin (GAS) and the activities of iNOS and COX-2 were also measured. The OD value was then measured on a microplate reader at 450 nm (BioTek Instruments, Inc., Winooski, VT). All tests were performed according to the manufacturer’s recommendation.

### Western blot analysis

Homogenized gastric tissue was lysed with radioimmunoprecipitation assay (RIPA) lysis buffer (Beyotime, Nanjing, China) containing protease and phosphatase inhibitors. Then, the solution was centrifuged at 12,000×*g* at 4 °C for 5 min. The supernatant was collected and concentrations were then determined using bicinchoninic acid (BCA) protein assay kit (Beyotime, Nanjing, China). Equal amounts of protein samples were separated by 8% sodium dodecyl sulphate-polyacrylamide gel electrophoresis (SDS-PAGE) and transferred onto polyvinylidene fluoride (PVDF) (MilliporeSigma, Burlington, MA) membranes. The PVDF membranes were blocked with 5% BSA for 1 h on a shaker, then incubated with 1:1000 dilution of primary anti-NF-κBp65 (Abcam, cat. no. Ab16502), COX-2 (Abcam, cat. no. ab15191), TNF-α (Abcam, cat. no. ab9739), iNOS (Abcam, cat. no. ab15323), IL-1β (Abcam, cat. no. Ab2105) and β-actin (Abcam, cat. no. Ab8245) at 4 °C overnight, and subsequently processed on a shaker at room temperature for 2 h by means of horseradish peroxidase-conjugated second antibody. Finally, these bands were visualized by a gel imaging system (Bio-Rad, Hercules, CA) using chemiluminescence detection reagents (Beyotime, Nanjing, China). Densitometrical analysis was performed using an Image J software (National Institutes of Health, Bethesda, MD).

### Statistical analysis

All experimental results were expressed as mean ± standard deviation and analysed with SPSS 25.0 (SPSS, Chicago, IL) using a one-way analysis of variance followed by Fisher's least significant difference (LSD) test for multiple comparisons. *p* Values <0.05 indicated statistical significance.

## Results

### Identification of F-AOH components

**Figure 1.**
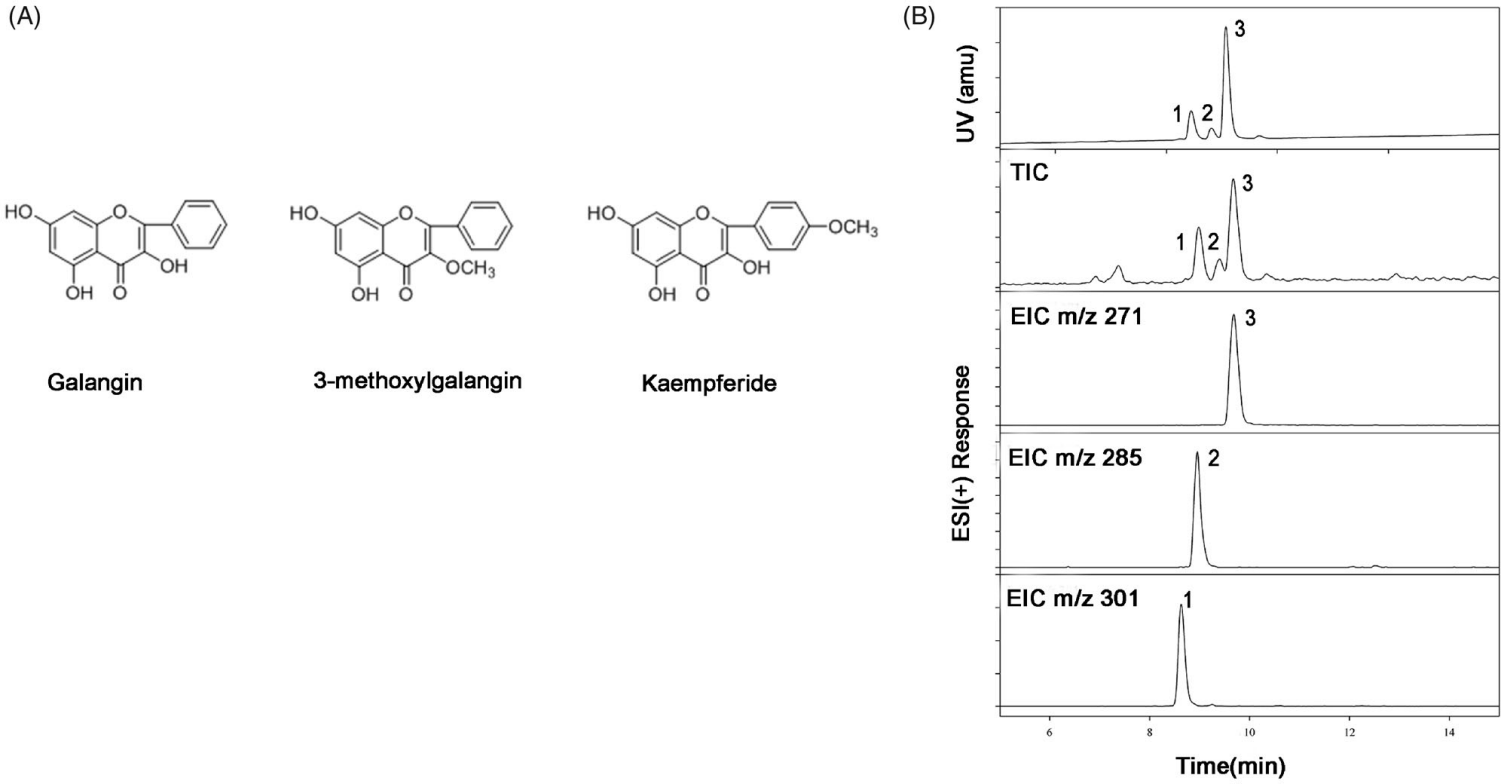
Chemical structures of the three major constituents in the flavonoid fraction. (A) Galangin: 3, 5, 7-trihydroxyflavone; 3-methyl Galangin: 5, 7-Dihydroxy-3-methoxyflavone; Kaempferide: 3, 5, 7-trihydroxy-4-methoxyfla-vone. (B) UHPLC-DAD-MS of F-AOH (1. Kaempferide; 2. 3-methyl Galangin; 3. Galangin).

Figure 1(A) presents the primary active ingredients of F-AOH. It was found that the flavonoids contained galangin (1), 3-methylgalangin (2) and kaempferide (3) (Figure 1(B)).

### General evaluation of gastric tissue

**Figure 2.**
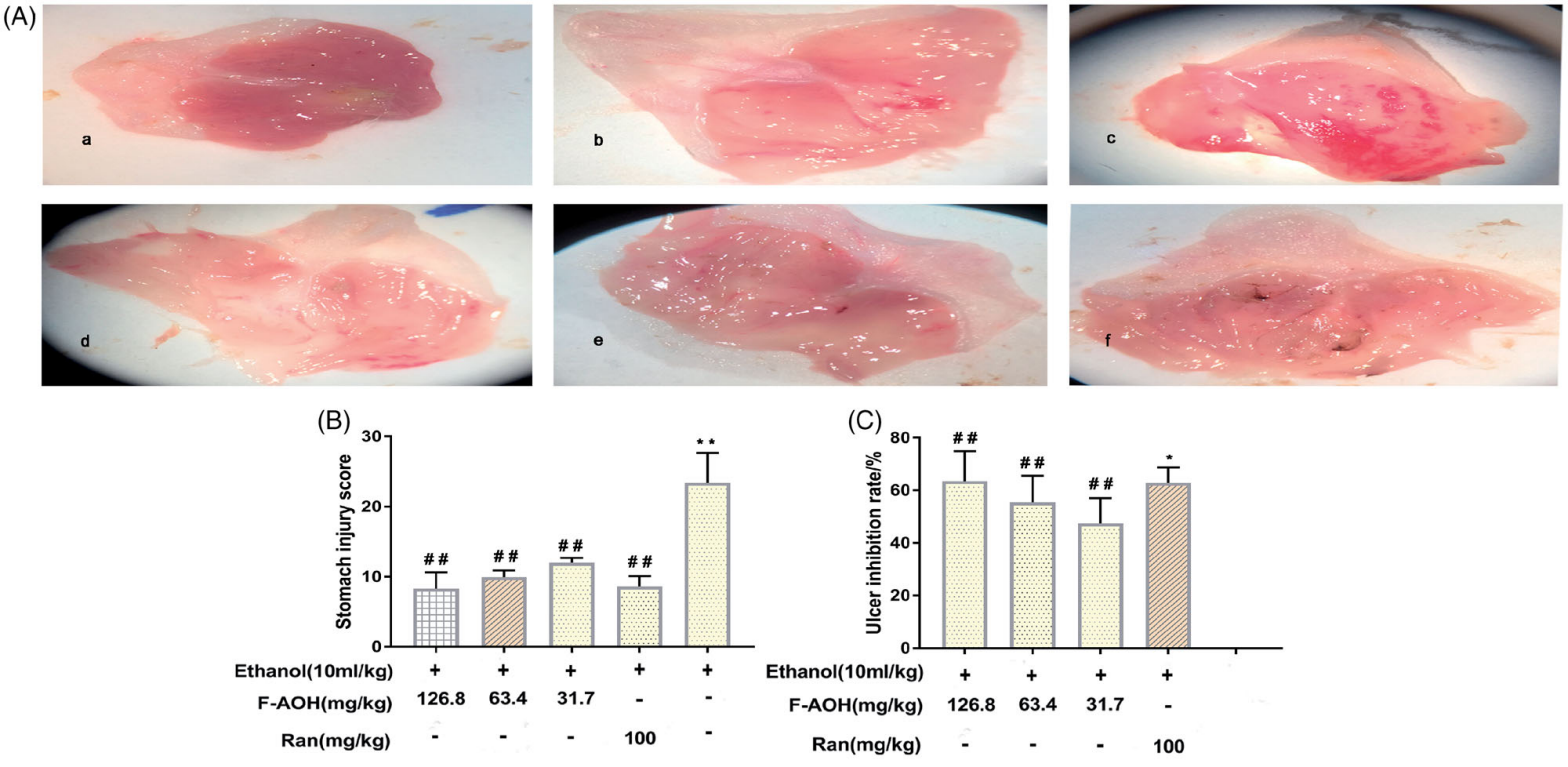
General evaluation of gastric tissue (A) Effects of F-AOH on ethanol-induced gastric mucosal injury. (a) Normal; (b) Ran:100 mg/kg; (c) Ethanol; (d) F-AOH (31.7 mg/kg); (e) F-AOH (63.4 mg/kg); (f) F-AOH (126.8 mg/kg). (B) Ulcer injury index in each group. Values are expressed as means ± standard deviation (*n* = 8). *p < 0.05, compared with normal group; #*p* < 0.05 and ##*p* < 0.01, compared with ethanol group. (C) Percentage of treatments group inhibition. *p < 0.05 compared with Ran group; ##*p* < 0.01 compared with ethanol group.

The normal group showed complete gastric mucosal structure without any signs of mucosal erosion. The ethanol group presented extensive haemorrhagic necrosis and ulcerative blood spot formation. Compared with the ethanol group, the groups with F-AOH (126.8, 63.4 and 31.7 mg/kg) or Ran (100 mg/kg) treatment had a significantly reduced degree of ethanol-induced gastric mucosal damage (Figure 2(A)). Figure 2(B) shows that the UI was significantly reduced in animals pre-treated with both F-AOH (126.8, 63.4 and 31.7 mg/kg) and Ran (100 mg/kg) (*p* < 0.01) as compared with that in the ethanol group. Figure 2(C) shows that the percentage of treatment group inhibition with both F-AOH (126.8, 63.4 and 31.7 mg/kg) and Ran (100 mg/kg) were 62.8, 47.5, 55.5 and 63.5%, respectively.

### Histopathological examination

**Figure 3.**
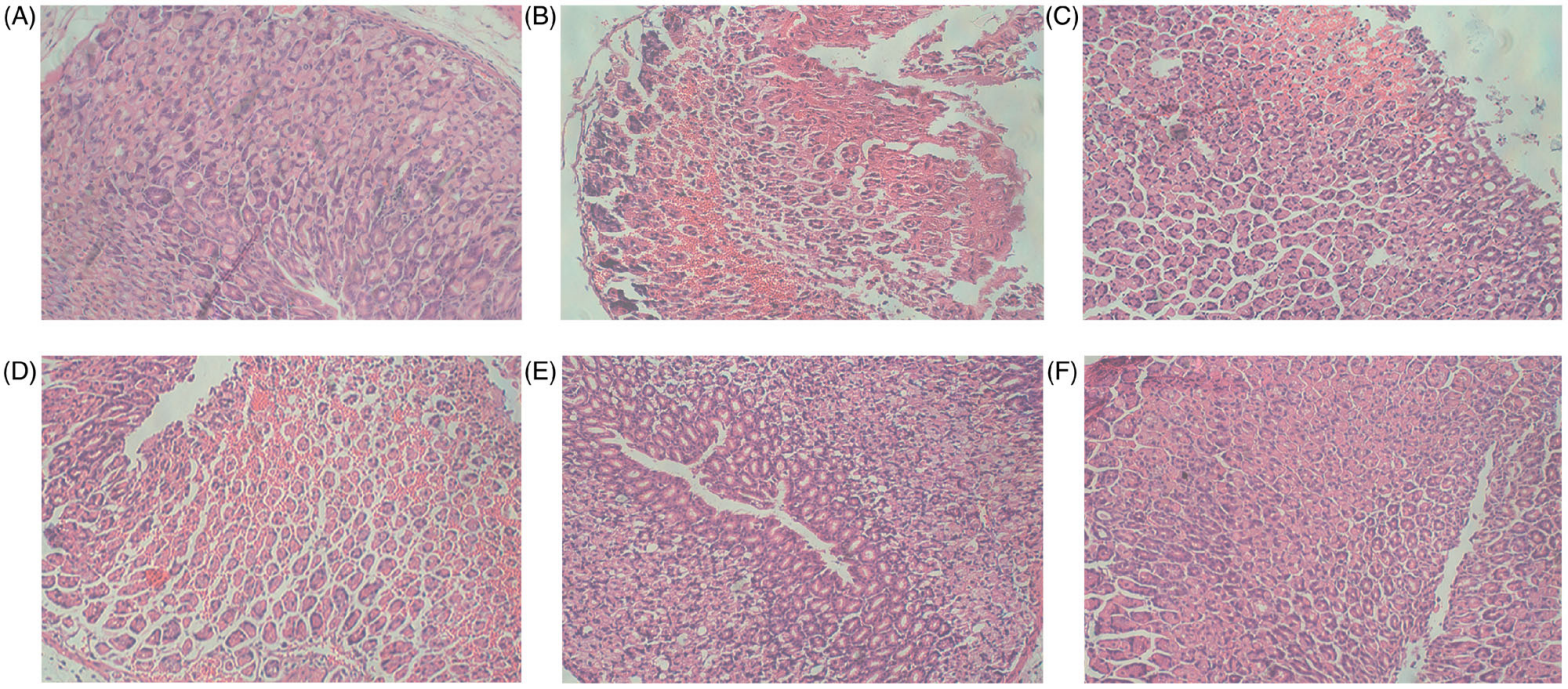
Histological evaluation of F-AOH on ethanol-induced gastric ulcer (H&E staining (magnification: ×400)) (A) Normal; (B) Ethanol; (C) Ran (100 mg/kg); (D) F-AOH (31.7 mg/kg); (E) F-AOH (63.4 mg/kg); (F) F-AOH (126.8 mg/kg).

The histopathological evaluation of gastric tissues (Figure 3) shows that the normal group did not undergo any significant histopathological changes, demonstrating complete tissue structure; neatly arranged somatic cells; and no gastric mucosal erosion, ulceration or tissue layer degeneration, necrosis, and inflammatory cell infiltration. In contrast, the ethanol group showed that the structural integrity of the gastric tissue was damaged, there was epithelial tissue loss, and there were ulceration and erosion on the gastric mucosa. In addition, the cells were randomly arranged and damaged, and inflammatory cell infiltration was observed in gastric tissue. Compared with the ethanol group, the groups with F-AOH (126.8, 63.4 and 31.7 mg/kg) or Ran (100 mg/kg) treatment had reduced gastric mucosal erosion and inflammatory cell infiltration.

### Determination of proinflammatory factors

**Figure 4.**
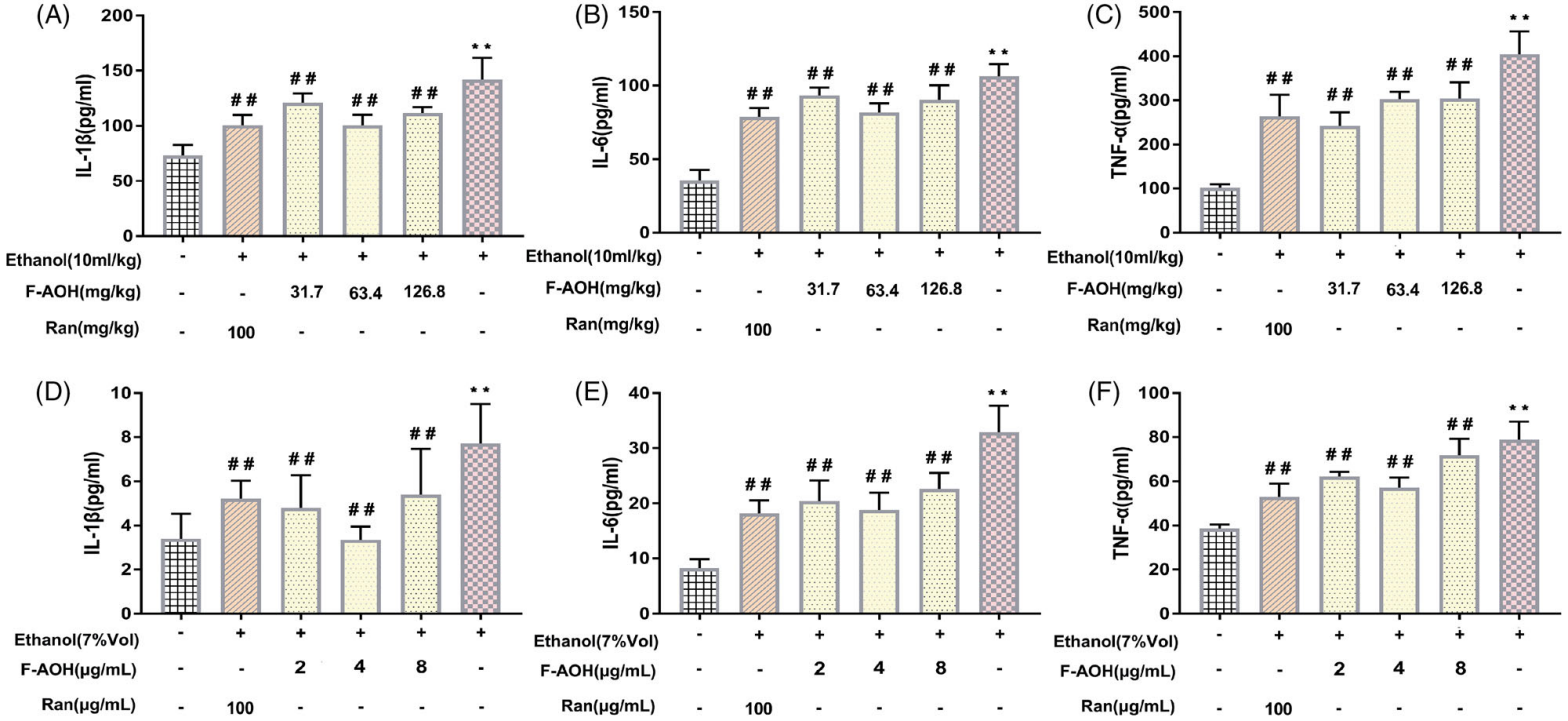
Effects of F-AOH on inflammatory factors in mice (A–C) and GES-1 cells (D–F). Values are expressed as means ± standard deviation (*n* = 6). *p < 0.05 and **p < 0.01 compared with normal group; #*p* < 0.05 and ##*p* < 0.01 compared with ethanol group.

Ethanol treatment in mice resulted in a significant increase in the levels of TNF-α, IL-1β and IL-6 as compared with those of the normal group (*p* < 0.01, Figure 4). In the meantime, treatment with F-AOH lowered the elevated levels of proinflammatory cytokines in gastric tissue (*p* < 0.01, Figure 4(A–C)) and in the cell supernatant (Figure 4(D–F)). F-AOH treatment at 63.4 mg/kg significantly reduced the expression of IL-1β, IL-6 and TNF-α when compared with Ran treatment (100 mg/kg) and F-AOH treatment at 126.8 and 31.7 mg/kg. In cell experiments, the inhibition of proinflammatory factor levels was optimally suppressed by F-AOH (4 μg/mL) (*p* < 0.01, Figure 4(D–F)). Compared with ethanol treatment, *in vitro*/*in vivo* experiments demonstrated that the treatment with F-AOH can inhibit the upregulation of TNF-α, IL-1β and IL-6 inflammatory factors (*p* < 0.01).

### Effect of F-AOH on the levels of MTL, GAS, PGE2 and NO and on the activities of iNOS and COX-2

**Figure 5.**
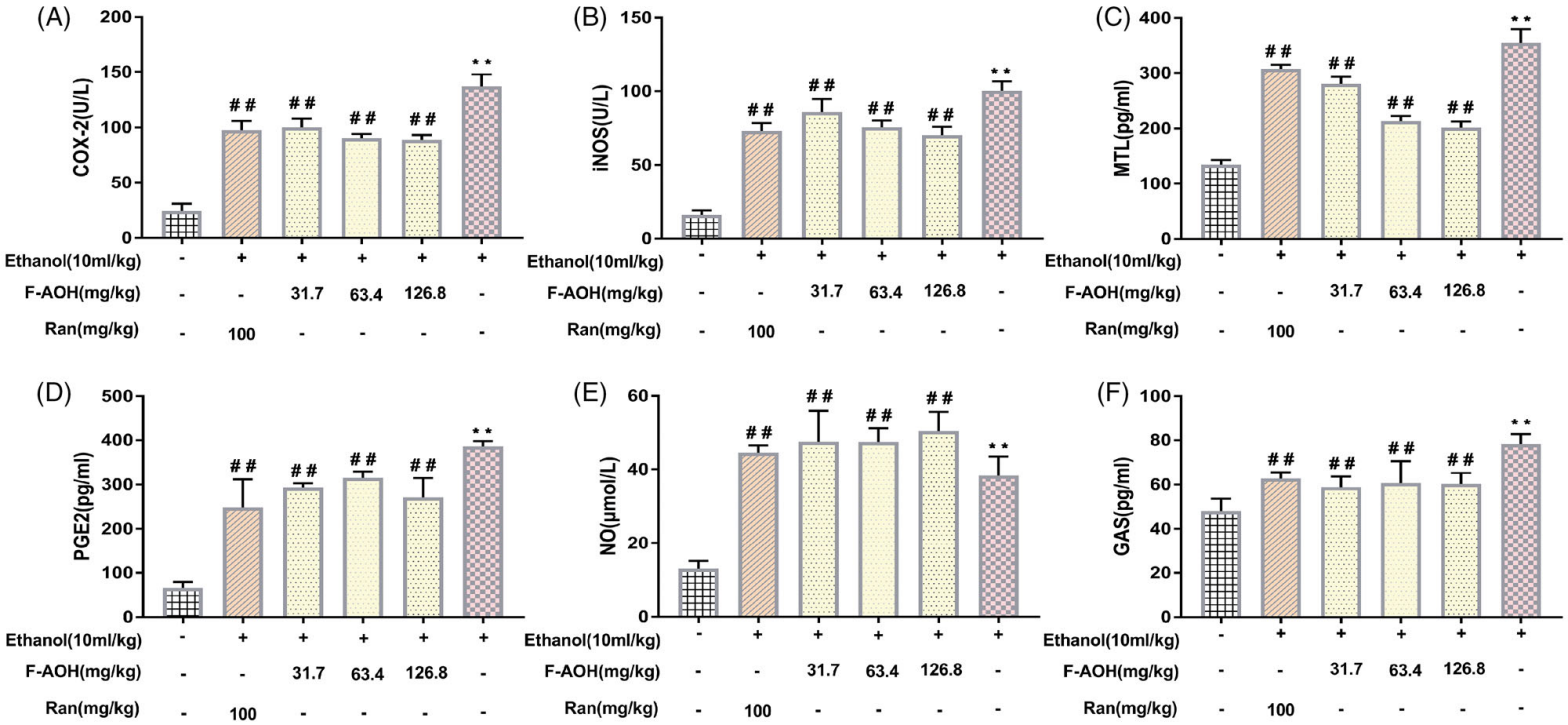
Effect of F-AOH on the levels of PGE2, MTL, GAS, NO and on the activities of iNOS and COX-2 (A–B) Effects of F-AOH on the activities of iNOS and COX-2. (C–F) Effects of F-AOH on the Levels of PGE2, NO, MTL, and GAS. Values are expressed as means ± standard deviation (*n* = 6). **p < 0.01 compared with normal group; #*p* < 0.05 and ##*p* < 0.01 compared with ethanol group.

Compared with the normal tissues, the levels of PGE2, MTL, GAS and NO and the activity of COX-2 and iNOS in ethanol-induced gastric tissues were significantly increased (*p* < 0.01, Figure 5). Treatment with F-AOH and Ran could lower the elevated activities of COX-2 and levels of PGE2 (*p* < 0.01, Figure 5(A,D)). F-AOH (63.4 and 126.8 mg/kg) significantly inhibited the production of MTL, when compared with Ran (100 mg/kg) and F-AOH treatment (31.7 mg/kg) (*p* < 0.01, Figure 5(C)). Treatment with F-AOH also inhibited the increase in GAS (*p* < 0.01, Figure 5(F)). In addition, F-AOH can also increase NO content and reduce iNOS activity (*p* < 0.05, Figure 5(B,E)).

### Cell activity value

**Figure 6.**
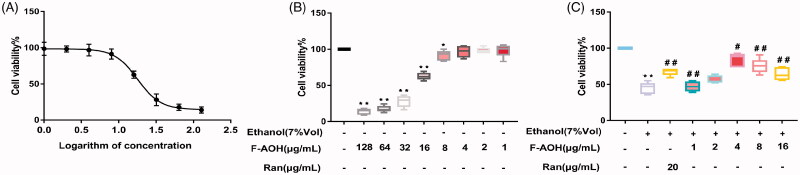
Defensive effects of F-AOH. (A) IC50 of F-AOH on the viability of GES-1 cells was examined. (B) Various concentrations of F-AOH (0, 1, 2, 8, and 16 μg/mL) were used to detect their effects on cell viability. (C) Various concentrations of F-AOH were used to attenuate ethanol-induced inhibition of cell viability. Values are expressed as means ± standard deviation (*n* = 6). *p < 0.05 compared with normal group; #*p* < 0.05 and ##*p* < 0.01 compared with ethanol group.

As shown in Figure 6(A), the 50% inhibitory concentration (IC_50_) of F-AOH in inducing the cellular damage model was 17 μg/mL. The cytotoxicity measurement of F-AOH showed that at concentration levels of 1, 2, 4 and 8 μg/mL, F-AOH had no significant difference in cell viability. However, at concentrations greater than 16 μg/mL, F-AOH significantly reduced cell viability compared to controls (*p* < 0.01, Figure 6(B)). As shown in Figure 6(C), the activity of the cells in the ethanol group was significantly decreased. Conversely, the loss of cell viability was significantly recovered with F-AOH treatment (*p* < 0.05 and *p* < 0.01); however, the trend of cell activity increased initially and then decreased with the increase of F-AOH concentration. When the concentration of F-AOH was controlled at 4 μg/mL, the cell activity was optimal. Interestingly, when the concentration of F-AOH is greater than that in the F-AOH group treated with 4 μg/mL, the cell activity decreases as the drug concentration increases. We believe that this may be related to the inhibition of cell activity by excessive drug concentration. Considering the dose factor and concentration limits, we selected the F-AOH groups treated with 8, 4 and 2 μg/mL as the drug group. The results showed that the active ingredient in F-AOH is effective against ethanol-induced gastric mucosal cell damage.

### NF-κBp65, COX-2, TNF-α, IL-1β and iNOS protein expression levels

**Figure 7.**
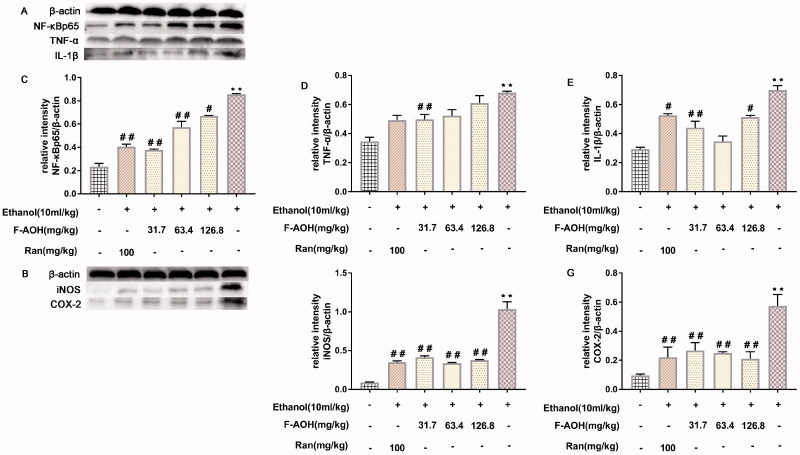
NF-κBp65, COX-2, TNF-α, IL-1β and iNOS protein expression levels (A-B) Effects of F-AOH on the protein expression of NF-κBp65, TNF-α, IL-1β, COX-2, and iNOS in gastric tissues. (C–G) Quantification of the relative protein levels of NF-κBp65, TNF-ℵ, IL-1β, COX-2, and iNOS. **p < 0.01 compared with ethanol group; #*p* < 0.05 and ##*p* < 0.01 compared with ethanol group.

As shown in Figure 7, the expression levels of several target proteins in the ethanol group were significantly higher than those in the normal group (*p* < 0.01). Compared with ethanol treatment, treatment with F-AOH blocked the ethanol-induced activation of NF-κBp65 (*p* < 0.01, Figure 7(A)), TNF-α (*p* < 0.01, Figure 7(B)), COX-2 (*p* < 0.01, Figure 7(C)) and iNOS (*p* < 0.01, Figure 7(D)). These results suggest that ethanol stimulation activates the NF-κB/COX-2 pathway and its downstream proteins, while F-AOH treatment blocks the activation of these proteins and reduces the inflammatory damage caused by ulcers.

## Discussion

The ethanol-induced gastric ulcer model has been widely used in the treatment of gastric ulcer and in the study of protective mechanisms of drugs. Subsequently, the mouse model of acute gastric ulcer was established in accordance with the ethanol-induced method to explore the effects of F-AOH on acute gastric ulcer. In addition, some previous studies reported that the extract of *A. officinarum* has anti-inflammatory activity (Jeong et al. 2008; Elgazar et al. 2018; Gong et al. 2018), which provides us with the theoretical basis of F-AOH treatment against gastric ulcer. Ethanol stimulation is associated with a sharp increase in the levels of proinflammatory factors in gastric tissue, congestion and oedema of gastric mucosa (Boutemine et al. 2018), death of epithelial cells, local or multiple haemorrhagic ulcers (Tarnawski et al. 1987), and tissue degeneration and necrosis (de Souza et al. 2019). These changes are consistent with our experimental results, and the pre-treatment of F-AOH significantly reduces these changes. These present results confirm the effectiveness of F-AOH on ethanol-induced gastric ulcer, thereby reducing the risk of gastric lesions by inhibition of gastric mucosal damage.

Proinflammatory cytokines, particularly TNF-α, IL-1β and IL-6, are closely associated with the degree of mucosal damage in gastric ulcer. TNF-α is an inflammatory cytokine secreted by macrophages. It is also the most important inflammatory factor in the early stage of gastric ulcer; it aggravates the degree of gastric ulcer by activating inflammatory-related cytokines and neutrophils migrating to gastric tissue (Fu et al. 2018) and inducing the activation of the NF-κB signalling pathway (Rozza et al. 2014). Studies have shown that the release and expression of IL-1β and IL-6 play an important role in gastrointestinal diseases such as gastric ulcer (Plebani et al. 1995), gastric cancer (Kabir et al. 1995) and colitis (Yamamoto et al. 2000; Wang et al. 2014). They could participate in the inflammatory process by upregulating the expression of endothelial cell adhesion molecules in tissues and serum and by inducing lymphocyte activation and differentiation, including the aggregation, activation and infiltration of inflammatory cells. Therefore, IL-1β and IL-6 levels can be used as indicators to evaluate the severity of ethanol-induced acute gastric ulcer, and so inhibiting the release of inflammatory cytokines is considered as one of the effective methods of reducing the severity of gastric mucosal injury. In our experiment, the levels of proinflammatory cytokines in gastric ulcer tissue and GES-1 cell supernatant were significantly increased in the ethanol-induced model. We found that the mucosa of gastric ulcer tissue was damaged; epithelial cells were decreased, destroyed or randomly arranged; and ulceration and erosion were formed in the tissue as confirmed by direct visualization and histopathological methods. However, the levels of TNF-α, IL-1β and IL-6 in tissue and GES-1 cell supernatant were significantly decreased, and the corresponding gastric ulcer and mucosal integrity were improved in the Ran and F-AOH groups. This finding demonstrates that F-AOH inhibits ethanol-induced gastric mucosal damage and local ulceration by inhibiting the production of proinflammatory factors.

NF-κB has been considered as a significant and essential transcription factor for the expression of various inflammatory mediators and plays an important role in regulating the body’s immune response. In our experiment, the stimulation of ethanol activated the immune system in mice and triggered cascade immune response in cells, causing phosphorylation and decomposition of the I-κB family; thus, the NF-κB was released from the I-κB/NF-κB complex, and significantly enhanced the expression levels of TNF-α, IL-1β and IL-6, which promote the expansion of inflammation in ulcer tissues. Furthermore, the administration of F-AOH effectively reduced the activation of NF-κB in tissues and inhibited the expansion of gastric ulcer. This finding is consistent with those of previous studies in that the inflammatory response and ulcer severity can be improved by regulating the correlation between NF-κB and the transcription of proinflammatory cytokines (Hui and Fangyu 2017).

As an antioxidant and a protective factor for the gastric mucosa, NO can protect the integrity of the gastric mucosa (Uno et al. 2004), and increase the oxygen supply by increasing the blood flow and microcirculation of the gastric mucosa (Takeuchi et al. 1997). Ethanol can induce NO release through iNOS overexpression and react with superoxide anion (O_2_^–^) to form peroxynitrite (ONOO^–^), which leads to lipid peroxidation damage of gastric tissue and accelerates gastric mucosal damage (Al-Quraishy et al. 2017). This is in agreement with that of our experimental results: the expression of NO in ethanol-induced gastric ulcer tissue is significantly increased. Interestingly, we found that F-AOH did not reduce the expression of NO, but continued to increase the level of NO. We believe that the underlying mechanism of F-AOH to promote the healing of gastric ulcers is to further increase the NO content on the basis of stimulating ethanol-induced iNOS production in the short term, thereby quickly completing the inflammatory response and contraction of ulcers, improving mucosal microcirculation, and effectively removing oxygen free (Elliott and Wallace 1998). In addition, in the early stage of physiologic inflammation, a low concentration of NO can activate the NF-κB pathway through its own amplification and the interaction of proinflammatory factors. When the concentration of NO increases abnormally, it can generate negative feedback to the NF-κB pathway and block its activation. At the same time, it can also lead to the suppression of the transcription of target genes related to NF-κB, including iNOS that promotes the increase of NO in the early stage, which in turn reduces NO and other inflammatory factors and accelerates the completion of the inflammatory response (Park et al. 1997). In our experiment on detecting iNOS activity and protein expression, we found that, compared with the model group, the iNOS activity and protein expression both decreased, indicating that the high concentration of NO produced in the early stage of inflammation will result in negative feedback towards iNOS and inhibit the expression of iNOS protein and its generation. This may also be considered as one of the molecular mechanisms of the anti-inflammatory effect of NO in the ethanol-induced injury model.

COX-2, an inducible enzyme that releases PGE2, mediates the body’s inflammatory response and is normally expressed at low levels. COX-2 plays a significant role in the development of gastric ulcers and is related to the pathology of gastric mucosa. PGE2 can promote the secretion of gastric mucus and bicarbonate, repair mucosal epithelial cells, improve microcirculation, and enhance the defence and repair capacity of the gastric mucosa (Sofidiya et al. 2015). There is evidence that the COX-2 promoter sequence contains two NF-κB binding sites, indicating that NF-κB as a COX-2 upstream signalling pathway plays a key role in the regulation and expression of COX-2 transcription level. Thus, F-AOH improves the mechanism of inhibiting ethanol-induced ulcer symptoms by blocking the NF-κB/COX-2/PGE2 signalling pathway.

As generally known, gastric acid is one of the key factors that play an important role in the development of gastric ulcer (Wijeratne et al. 2015). GAS can promote the synthesis of gastric acid and pepsin, stimulate the vagus nerve, and enhance the physiological function of gastric motility. MTL can promote gastric motility, smooth muscle contraction and gastric emptying. In our study, GAS and MTL levels increased significantly in the ethanol group, indicating that ethanol-induced ulcer could block the physiological feedback of GAS and MTL in the stomach and aggravate the degree of gastric mucosal injury through the increase of both levels in gastric tissues. However, the levels of GAS and MTL decreased significantly in gastric tissues after pre-treatment with F-AOH and Ran, suggesting that F-AOH can affect the secretion of GAS and MTL, thereby improving gastric acid and gastrointestinal motility and promoting ulcer healing.

## Conclusions

The results showed that F-AOH had anti-inflammatory and protective effects on ethanol-induced gastric ulcer. The underlying mechanism may be related to the abnormally increased negative feedback regulation of NO, the activation of NF-κB/COX-2 signalling pathway, and the influence of downstream inflammatory factor levels. These findings provide a basis for the clinical treatment of gastric ulcer with the Chinese herbal medicine galangal, demonstrating the pharmacological activity and anti-inflammatory effect of F-AOH, which can be used as a potential gastroprotective agent. Furthermore, with regard to the anti-inflammatory and protective effects of F-AOH on gastric ulcers, further studies are required to determine the exact mechanism of the feedback effect of NO on the biphasic regulation between the NF-κB/COX-2 and downstream signalling pathways.
